# Factors Associated With Mortality During the First Year Post Infarction: Survival Analysis of Patients With Acute Myocardial Infarction in Colombia

**DOI:** 10.7759/cureus.58118

**Published:** 2024-04-12

**Authors:** Anderson Bermon, Maricel Licht-Ardila, Fabián Manrique-Hernández, Alexandra Hurtado-Ortiz, Diana Cañon, Carlos Federico Molina Castaño

**Affiliations:** 1 Epidemiology, Escuela de Graduados, Universidad CES, Medellin, COL; 2 Epidemiology, Fundación Cardiovascular de Colombia, Piedecuesta, COL; 3 Epidemiology and Public Health, Fundación Cardiovascular de Colombia, Piedecuesta, COL; 4 Cardiology, Fundación Cardiovascular de Colombia, Piedecuesta, COL; 5 Epidemiology, Tecnológico de Antioquia - Institución Universitaria, Medellin, COL

**Keywords:** chronic kidney disease, risk factors, mortality, acute myocardial infarction, cardiovascular diseases

## Abstract

Introduction: Cardiovascular diseases account for over 80% of global deaths. Risk factors and social determinants influence mortality in patients post acute myocardial infarction (AMI).

Objective: To evaluate factors associated with post-AMI mortality during the one-year follow-up.

Materials and methods: The study is a prospective cohort study of adults aged 18 years and older with type 1 AMI conducted between October 2021 and January 2024. Intrahospital and outpatient information was collected. Statistical analyses included the Kaplan-Meier survival curve and Cox regression analysis. Proportional hazards and model predictive capacity were evaluated.

Results: A total of 1873 patients were included, with a 9.4% mortality rate in the first year. At one year, the estimated survival probability was 88.61% (95% CI: 86.82-90.18). Cox analysis identified several factors associated with mortality, highlighting age (HR = 1.04, 95% CI: 1.02-1.06, p = 0.001), diabetes (HR = 1.77, 95% CI: 1.09-2.87, p = 0.020), renal insufficiency (HR = 2.25, 95% CI: 1.32-3.84, p = 0.003), and type of intervention. The model evaluation showed strong predictive capacity.

Conclusions: It is essential to emphasize the importance of comprehensive management in AMI patients with risk factors such as diabetes and chronic kidney disease, as they are significant predictors of mortality during the first year post infarction.

## Introduction

At the global level, it has been reported that more than four out of every five deaths from cardiovascular disease (CVD) are attributed to heart attacks and strokes [1]. In Colombia, the prevalence of ischemic heart disease in 2019 was 0.57%. The highest prevalence was observed among individuals aged over 80 years at 6.99%, followed by those aged 75-79 years at 5.18% and 70-74 years at 4.14% [2]. Despite significant advancements in the treatment and prognosis of acute myocardial infarction (AMI) in recent decades, ischemic heart disease was responsible for two million deaths in 2019 [3]. However, it remains one of the leading causes of mortality worldwide, posing a significant burden for both society and affected individuals [4].

Previously, the majority of deaths were limited to the elderly population. However, nowadays, changes in the lifestyles of younger individuals have led to an increase in the prevalence of atherosclerosis at earlier ages, resulting in a significant rise in premature cardiovascular events and a higher risk of mortality [5]. Furthermore, risk factors such as diabetes, hypertension, peripheral arterial disease, chronic kidney disease (CKD), and a history of cardiovascular events are associated with adverse outcomes following an AMI [6].

On the other hand, the nutritional index could serve as a predictor to identify patients at high risk of all-cause mortality one year after experiencing an AMI [7]. Likewise, it is relevant to note that there is a gender disparity in this context, with this disease burden trend being more pronounced in women compared to men [8]. However, the prognosis in women with non-ST segment elevation myocardial infarction (NSTEMI) tends to be more favorable, in contrast to higher early mortality following an ST-segment elevation myocardial infarction (STEMI). Moreover, there is evidence indicating a decrease in mortality rates among individuals with STEMI in recent times. This trend could be linked to improvements in pharmacotherapy, increased availability of primary percutaneous coronary intervention (PCI), and the formulation of international clinical guidelines for the management of STEMI [9].

Similarly, socioeconomic factors such as lack of education, poverty, and income inequality are crucial social determinants of cardiovascular health [10]. Individuals with low incomes face a higher risk of experiencing various adverse health outcomes, including AMI, reinfarction, and mortality from coronary artery disease [11]. Although the reasons behind this disparity may include differences in smoking, hypertension, and diabetes, it has been observed to persist even after adjusting for these cardiac risk factors [12]. Therefore, it is essential to comprehensively understand and address CVD-related mortality, especially in patients who have experienced an AMI. Hence, the primary objective of this study is to evaluate the factors associated with mortality during the first year following AMI.

## Materials and methods

**Figure 1:**
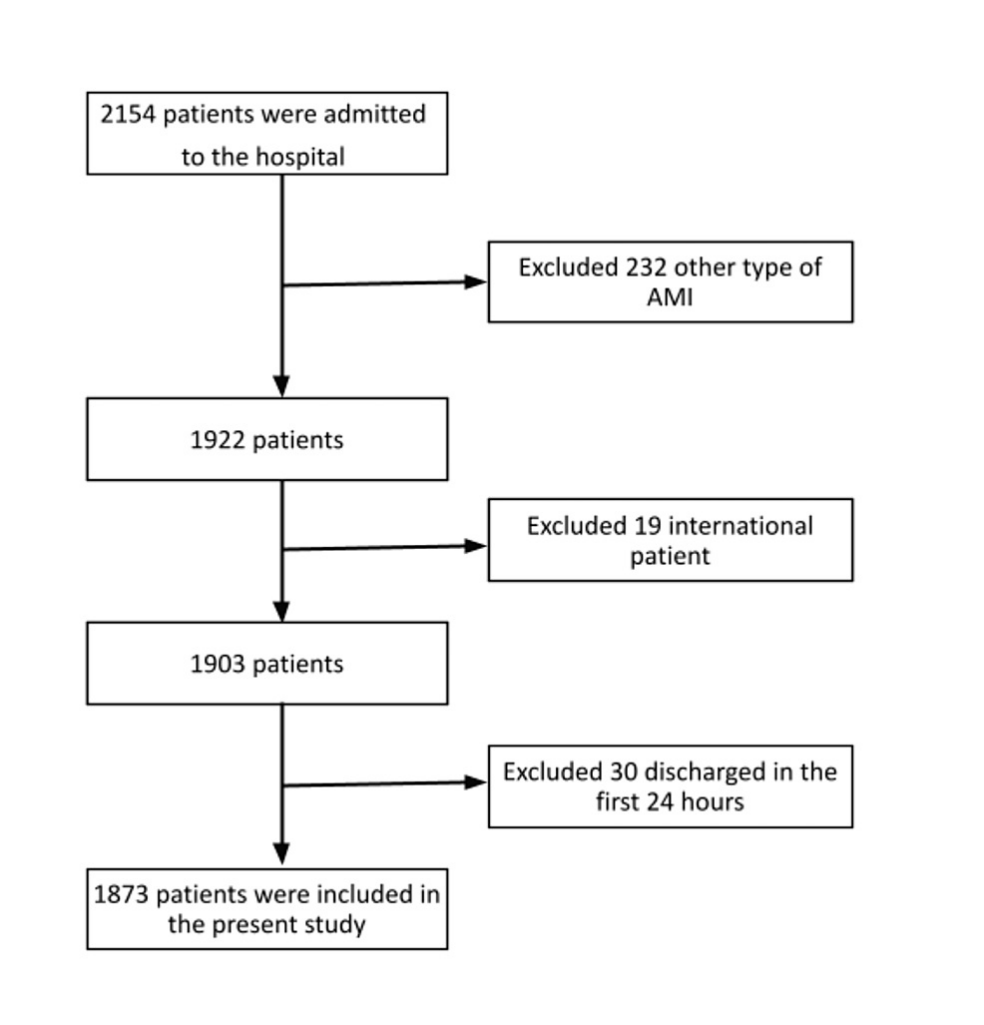
Algorithm for inclusion of patients with acute myocardial infarction (AMI) type 1

A prospective cohort study was conducted on patients over 18 years old with type 1 AMI, during the period from October 2021 to January 2024. The patients admitted to a high-complexity institution, a referral center for cardiovascular disease in northeastern Colombia, were included. The variables were collected from both in-hospital stay and outpatient follow-up. Follow-up was conducted through telephone calls at 72 hours, two months, six months, and one year after discharge, with an in-person visit after the first month. Sociodemographic variables such as age, sex, marital status, education level, and area of origin were recorded, along with clinical and paraclinical variables, including medical history, smoking status, body mass index, type of AMI (STEMI and NSTEMI), risk scales (Global Registry of Acute Coronary Events (GRACE), Thrombolysis in Myocardial Infarction (TIMI), and Killip), premature AMI (which generally refers to AMI in men ≤ 55 years old or women ≤ 65 years old) [13], laboratory tests, type of intervention (medical treatment, PCI, and coronary artery bypass grafting (CABG)), and length of hospital stay. The outcome assessed was any-cause mortality, whether during hospitalization or during outpatient follow-up. International patients were excluded due to limitations in follow-up, as were those with stays of less than 24 hours and those discharged against medical advice. Follow-up continued upon readmission following the timeline of the initial institutional admission (Figure 1).

In the statistical analyses conducted, continuous variables were described using central tendency and dispersion, while categorical variables were shown through absolute frequencies and percentages. A bivariate analysis was performed for each independent variable using chi-square and Mann-Whitney U tests for categorical and continuous variables, respectively. Kaplan-Meier survival curves were constructed to evaluate patient survival one year after the coronary event. Differences between the groups were checked using the log-rank test. Additionally, Cox regression analyses were done, and the Breslow method was applied to handle tied values. Furthermore, the proportionality of hazards of the covariates was checked using Schoenfeld residuals to assess whether their effect over time remained constant. All of these steps were undertaken to examine the association between various independent variables and mortality. These survival analyses enabled the evaluation of the temporal relationship between explanatory variables and the outcome over time. Hazard ratios were calculated to quantify the risk, and statistical significance tests were performed, considering a level of p < 0.05. Finally, the predictive capacity of the model was evaluated using Harrell's C statistic. The data were collected in the Research Electronic Data Capture (REDCap, Vanderbilt University, Nashville, TN) [14] database under project PID 210. This platform is filled out by trained healthcare personnel, who daily input clinical history information for intrahospital data. Additionally, follow-up information is obtained through phone calls and recorded in REDCap. The analyses were carried out in STATA 16 (StataCorp LLC, College Station, TX).

This project has been approved by the institutional ethics committee and was conducted by the researchers in strict adherence to ethical standards at both the national and international levels in the field of scientific research.

## Results

The study included a total of 1873 patients, of whom 176 died in the first year post AMI, representing 9.4% of the sample. Losses to follow-up were less than 1%. The median age of deceased patients was significantly higher than that of living patients (73.5 years vs. 66 years, p < 0.001). Similarly, a higher percentage of women was observed among deceased patients compared to the living ones (38.64% vs. 29.40%, p = 0.011). Regarding marital status, significant differences were observed between the groups, with a higher percentage of single patients among the deceased (44.89% vs. 32.78%, p < 0.001), while the percentage of married or cohabiting patients was higher among the living ones (65.00% vs. 50.57%, p < 0.001).

Living patients showed a higher percentage of individuals with some level of education compared to the deceased (90.98% vs. 85.23%, p = 0.013). On the other hand, in the group of individuals who died, a higher percentage of comorbidities, such as diabetes (44.89% vs. 32.59%, p = 0.001), coronary artery disease (9.43% vs. 15.34%, p = 0.013), and CKD (6.48% vs. 23.86%, p < 0.001), was observed. However, dyslipidemia was higher in living patients (34.30% vs. 25.57%, p = 0.020). Nutritional status behaved differently between the groups, with those underweight being more prevalent among the deceased (9.79% vs. 15.74%, p < 0.001). Regarding tobacco and alcohol consumption, no significant differences were observed.

Regarding the type of heart attack, firstly, it was found that 40.90% of living patients experienced a STEMI, while this percentage was slightly higher, at 44.57%, among deceased patients. On the other hand, 59.10% of living patients presented with an NSTEMI. However, differences in the distribution of types of AMI between the groups were not statistically significant (p = 0.347). Premature infarction was found to be more frequent among the deceased (22.22% vs. 5.68%, p < 0.001). Additionally, 53.10% (p = 0.001) of deceased patients had three or more vessels involved.

Risk scales such as GRACE, TIMI, and Killip were analyzed, revealing significant differences between the groups, with deceased patients showing higher mortality risk and more complexity. Specifically, among patients classified as Killip IV, 43% died. The majority of living patients underwent PCI (this category refers to both primary and rescue), accounting for 67.45%, while among deceased patients, this proportion was slightly lower, at 57.39%. CABG was performed in 21.58% of living patients and 6.82% of deceased patients. These differences were statistically significant (p < 0.001).

The median troponin levels among deceased patients were higher (3830.6 ng/dL vs. 2182.9 ng/dL; p = 0.045). Similarly, creatinine levels were higher in the deceased group (1.25 mg/dL vs. 0.92 mg/dL, p < 0.001). Regarding intrahospital stay, the median was four days among living patients (interquartile range (IQR): 2-10 days), while among deceased patients, it was five days (IQR: 3-10 days) (p = 0.003) (Table 1).

**Table 1:** Sociodemographic and clinical characteristics of patients with acute myocardial infarction * Median (interquartile range). ** Refers exclusively to medical treatment, that is, PCI/CABG is not performed. *** PCI: percutaneous coronary intervention. **** CABG: coronary artery bypass grafting. GRACE: Global Registry of Acute Coronary Events; TIMI: Thrombolysis in Myocardial Infarction.

Characteristics	Categories	Alive (n = 1697)		Deceased (n = 176)		p
		n (%)	95% CI	n (%)	95% CI	
Age (years)	Median (IQR)*	66 (58-74)		73.5 (65-82)		<0.001
Gender	Female	499 (29.40)	27.28-31.61	68 (38.64)	31.68-46.08	0.011
Marital status						<0.001
	Single	556 (32.78)	30.58-35.05	79 (44.89)	37.65-52.34	
	Married or cohabiting	1102 (65.00)	62.67-67.21	89 (50.57)	43.17-57.93	
Education	Some level of education	1544 (90.98)	89.52-92.25	150 (85.23)	79.13-89.76	0.013
Place of residence	Rural area	320 (18.88)	17.08-20.81	40 (22.73)	17.09-29.55	0.218
	City	1375 (81.12)	79.18-82.91	136 (77.27)	70.44-82.90	
Clinical history	Hypertension	1143 (67.35)	65.08-69.54	124 (70.45)	63.25-76.76	0.403
	Diabetes	553 (32.59)	30.39-34.85	79 (44.89)	37.65-52.34	0.001
	Dyslipidemia	582 (34.30)	32.07-36.59	45 (25.57)	19.63-32.57	0.020
	Coronary artery disease	160 (9.43)	8.12-10.91	27 (15.34)	10.70-21.49	0.013
	Chronic kidney disease	110 (6.48)	5.40-7.75	42 (23.86)	18.10-30.76	<0.001
Nutritional status						<0.001
	Obesity	239 (14.09)	12.51-15.83	21 (11.93)	7.88-17.65	
	Overweight	597 (35.20)	32.96-37.50	33 (18.75)	13.61-25.25	
	Normal	694 (40.92)	38.60-43.28	95 (53.98)	46.52-61.25	
	Underweight	166 (9.79)	8.46-11.29	27 (15.34)	10.70-21.49	
Smoking	Yes	217 (12.79)	11.27-14.46	18 (10.23)	6.51-15.69	0.329
Alcoholism	Yes	97 (5.72)	4.70-6.92	6 (3.41)	1.53-7.41	0.201
Myocardial infarction type						0.347
	STEMI	694 (40.90)	38.57-43.25	78 (44.57)	37.32-52.05	
	NSTEMI	1003 (59.10)	56.74-61.42	97 (55.43)	47.94-62.67	
Premature myocardial infarction	yes	377 (22.22)	20.29-24.25	10 (5.68)	3.07-10.27	<0.001
Involved vessels #	>=3	573 (37.97)	35.55-40.45	60 (53.10)	43.79-62.18	0.001
TIMI score	Median (IQR)*	3 (2-4)		4 (3-5)		<0.001
GRACE score	Median (IQR)*	115.5 (96-135)		143 (120-171)		<0.001
Killip						<0.001
	I	1218 (83.65)	81.66-85.46	74 (47.74)	39.93-55.65	
	II	129 (8.86)	7.50-10.43	36 (23.23)	17.20-30.57	
	III	79 (5.43)	4.37-6.71	22 (14.19)	9.50-20.67	
	IV	30 (2.06)	1.44-2.93	23 (14.84)	10.03-21.40	
Type of treatment						
	Medical treatment**	186 (10.97)	9.56-12.54	63 (35.80)	29.01-43.19	<0.001
	PCI***	1144 (67.45)	65.18-69.64	101 (57.39)	49.91-63.53	
	CABG****	366 (21.58)	19.68-23.60	12 (6.82)	3.89-11.66	
Troponin I (ng/l)	Median (IQR)*	2182.9 (399-14414.3)		3830.6 (757-14165)		0.045
Creatinine (mg/dl)	Median (IQR)*	0.92 (0.78-1.14)		1.25 (0.93-1.8)		<0.001
Length of stay (days)	Median (IQR)*	4 (2-10)		5 (3-10)		0.003

**Figure 2:**
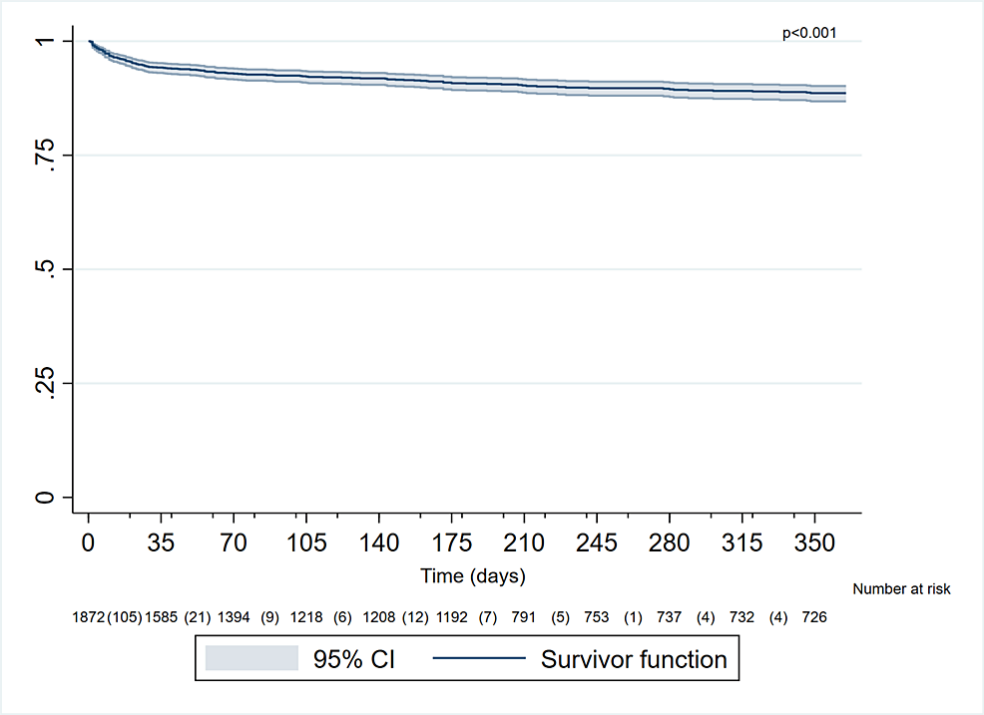
Kaplan–Meier estimates of the probability of death from any cause in 1873 subjects with acute myocardial infarction type 1

Figure 2 displays Kaplan-Meier estimates of survival probability for patients with AMI during the first year post event. At 30 days, the probability of being alive was 94.34% (95% CI: 93.18-95.31). At 90 days, survival was 92.46% (95% CI: 91.12-93.60), decreasing to 90.79% (95% CI: 89.28-92.09) at six months. Finally, at one year, the estimated survival probability was 88.61% (95% CI: 86.82-90.18).

**Figure 3:**
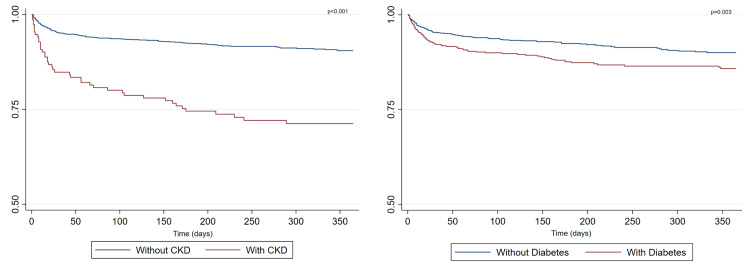
Kaplan-Meier estimates of the probability of death from any cause in subjects with acute myocardial infarction type 1 and comorbidities. CKD: chronic kidney disease.

Figure 3 depicts the one-year survival post AMI in patients with a history of diabetes and CKD. In nondiabetic patients, the probability of survival was 90.04% (95% CI: 87.83-91.86), whereas in diabetic patients, it was 85.81% (95% CI: 82.47-88.56). Similarly, the survival rate was 90.47% (95% CI: 88.69-91.98) in patients without CKD and 71.26% (95% CI: 63.12-77.92) in patients with CKD.

**Figure 4:**
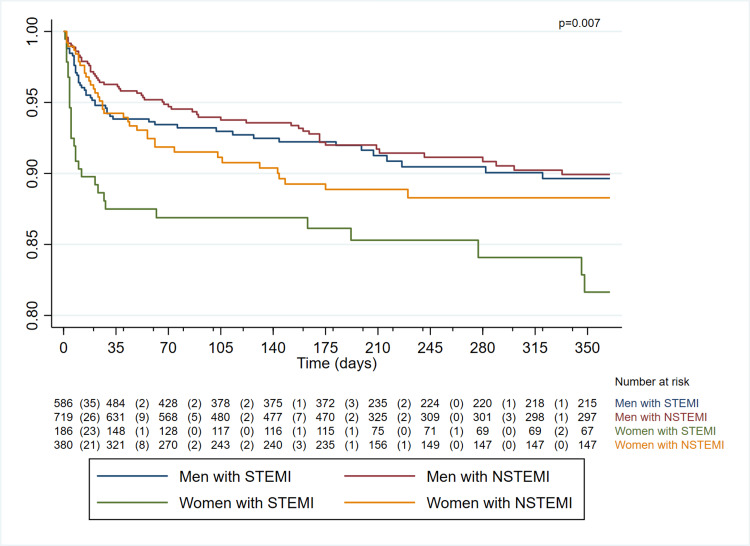
Follow-up of patients with acute myocardial infarction in the first year post event STEMI: ST-segment elevation myocardial infarction; NSTEMI: non-ST segment elevation myocardial infarction.

A one-year survival analysis stratified by gender and type of infarction was conducted, revealing that women and those with STEMI exhibit lower survival rates compared to men (81.64% (95% CI: 74.09-87.18) vs. 89.64% (95% CI: 86.30-92.21)). Regarding women with NSTEMI, initial survival rates were similar during the first 15 days, although over time, the probability of survival also decreased compared to that of men (88.28% (95% CI: 84.14-91.40) vs. 89.93% (95% CI: 86.99-92.23)) (Figure 4).

In the Cox analysis, adjusted for covariates, several factors associated with the risk of mortality in patients with AMI were identified. An increased risk was observed with age (HR = 1.04, 95% CI: 1.02-1.06, p = 0.001). Similarly, a history of diabetes mellitus (HR = 1.77, 95% CI: 1.09-2.87, p = 0.020) and CKD (HR = 2.25, 95% CI: 1.32-3.84, p = 0.003) showed significant associations with a higher risk of mortality. Additionally, risk scores such as TIMI and Killip scales were significant predictors of mortality. CABG was associated with a reduced risk of mortality compared to medical treatment or PCI (HR = 0.30, 95% CI: 0.13-0.68, p = 0.004). Similarly, the involvement of three or more vessels was related to an increased risk of mortality (HR = 2.05, 95% CI: 1.27-3.31, p = 0.003) (Table 2).

**Table 2:** Results of the Cox proportional-hazards model comparing mortality from any cause in 1873 subjects with type 1 acute myocardial infarction during one-year follow-up CABG: coronary artery bypass grafting; TIMI: Thrombolysis in Myocardial Infarction.

Characteristics	Hazard ratio	p	95% confidence interval
Age (years)	1.03981	0.001	1.0157 - 1.0644
Gender	1.20053	0.466	0.7343 - 1.9626
Diabetes	1.77241	0.020	1.0940 - 2.8714
History of chronic kidney disease	2.25104	0.003	1.3192 - 3.8412
TIMI score	1.22605	0.001	1.0825 - 1.3885
Killip	1.74464	<0.001	1.3950 - 2.1818
CABG	0.29850	0.004	0.1317 - 0.6767
Involved vessels #	2.04962	0.003	1.2678 - 3.3135

The assessment of model concordance was performed using Harrell's C index, revealing a Somers' D value of 0.70. This outcome indicates a robust predictive capability of the model, suggesting an effective distinction among variables associated with mortality.

## Discussion

The results of our study reveal that the overall one-year survival for both sexes is 88.61%, similar to findings reported in a survival study [15]. Kaplan-Meier survival analysis further reinforces the ongoing risk of mortality post AMI, with decreasing survival probabilities observed during the one-year follow-up period. Several significant factors associated with the risk of mortality after an AMI stand out. Age emerged as a relevant predictor, consistent with findings by Plakht et al., showing a steady increase in mortality risk with each additional year [16]. Furthermore, we found that certain risk factors, such as a history of diabetes mellitus and CKD, were significantly associated with an increased mortality risk [17].

Risk scores on the TIMI scale and Killip classification may serve as useful predictors of mortality after an AMI, with higher mortality risk associated with greater severity of congestive heart failure at presentation [18,19]. It is noteworthy that the type of intervention, specifically CABG, was identified as a significant predictor of lower mortality risk [20]. Likewise, a greater number of vessels involved may be associated with a higher mortality risk after an AMI, although no significant differences were found in the multivariate model regarding the type of infarction, STEMI or NSTEMI, differing from findings by Danchin [21], suggesting the importance of early and appropriate intervention in AMI patients.

Higher mortality was observed in women, consistent with findings by Sadowski et al. [22]. Additionally, higher levels of troponin and creatinine may be associated with increased mortality risk [23], and premature infarction may be an important predictor of mortality after an AMI. A longer hospital stay was associated in the bivariate analysis with a higher mortality risk, potentially explained by these patients having more severe disease or additional complications, influencing their prognosis and increasing the risk of mortality; however, these variables were not significant in the multivariate analysis.

Regarding social determinants, such as educational level, and their potential influence on the prognosis of patients following an acute coronary event, significant associations were observed in the bivariate analysis. However, after adjusting for various variables in the present study, these associations lost significance. Similarly, marital status could be linked to post-AMI prognosis, with a higher proportion of unmarried patients dying compared to married or cohabiting individuals, suggesting a possible implication of lack of social support. Although this finding was significant in the bivariate analysis, it did not maintain statistical significance after relevant adjustments [12]. These findings highlight the multifactorial complexity of post-AMI prognosis and underscore the need for a holistic evaluation of multiple clinical factors to guide the optimal management of these patients.

The strengths of this study are rooted in its meticulous methodology and thorough analysis. A prospective cohort design was utilized, guaranteeing the acquisition of robust data across a substantial duration. Additionally, the study benefited from a considerable sample size, enabling a comprehensive evaluation of demographic and clinical variables.

Within the study design, some limitations include loss to follow-up, which could potentially bias mortality estimates. However, to mitigate this potential bias, multiple contacts and follow-ups were conducted to minimize losses, along with the implementation of national mortality registry reviews.

Confounding factors

Despite adjusting for various variables, unmeasured confounding factors may still exist that could influence the observed associations between predictor variables and mortality outcomes.

Data quality

The accuracy and completeness of collected data, particularly from medical records, could affect result validity; however, data collection followed protocols and guidelines for third-party data validation.

Geographical bias

Study findings may be influenced by population demographics and healthcare systems within the specific geographic region where the study was conducted, limiting generalizability to other populations. However, the results align with those reported in the literature for other populations.

## Conclusions

In this study, age, diabetes, CKD, and involvement of multiple vessels are independent predictors of mortality, highlighting the multifactorial nature of post-AMI mortality risk. Although significant associations with social determinants were initially observed, their relevance diminished after adjusting for other covariates. Also, a potential link between the extent of myocardial injury with the severity of renal dysfunction and mortality is found. Understanding these factors can inform risk stratification and guide therapeutic decisions, underscoring the necessity of tailored approaches to enhance outcomes in AMI patients.
